# Supplementary material for: Modifications on the Tetrahydroquinoline Scaffold Targeting a Phenylalanine Cluster on GPER as Antiproliferative Compounds against Renal, Liver and Pancreatic Cancer Cells
Source: Pharmaceuticals (Basel). 2021 Jan 10;14(1):49. doi: 10.3390/ph14010049 (PMC7826836; doi:10.3390/ph14010049)
Supplement: Supplementary file 1 [file pharmaceuticals-14-00049-s001.pdf]

Supplementary Material.

^{1}H and ^{13}C NMR spectra data

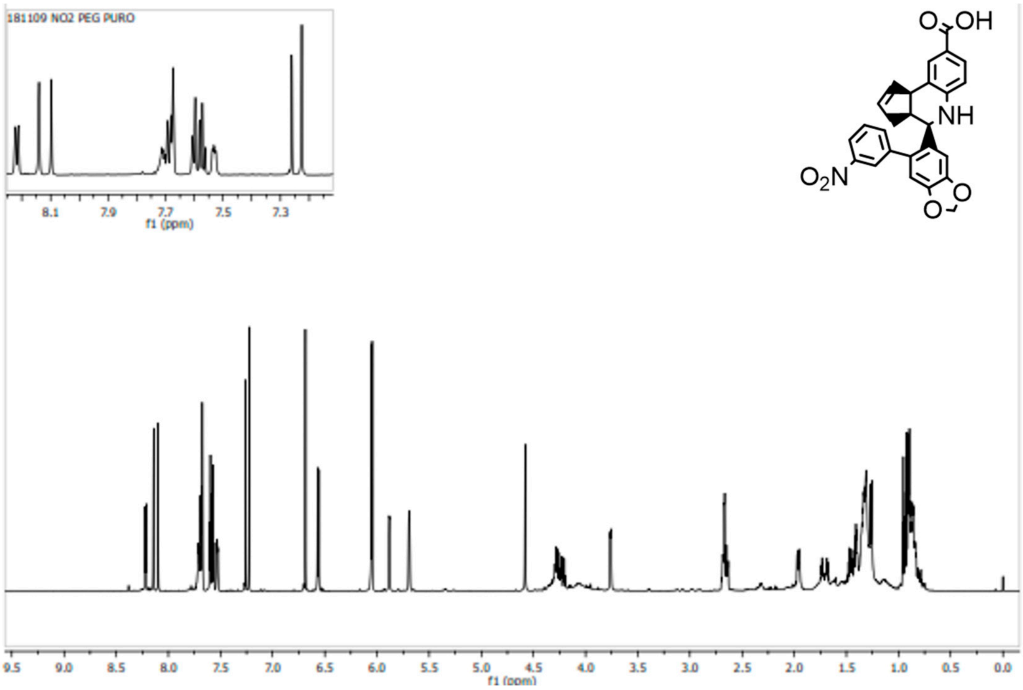

^{1}H NMR compound **4.**

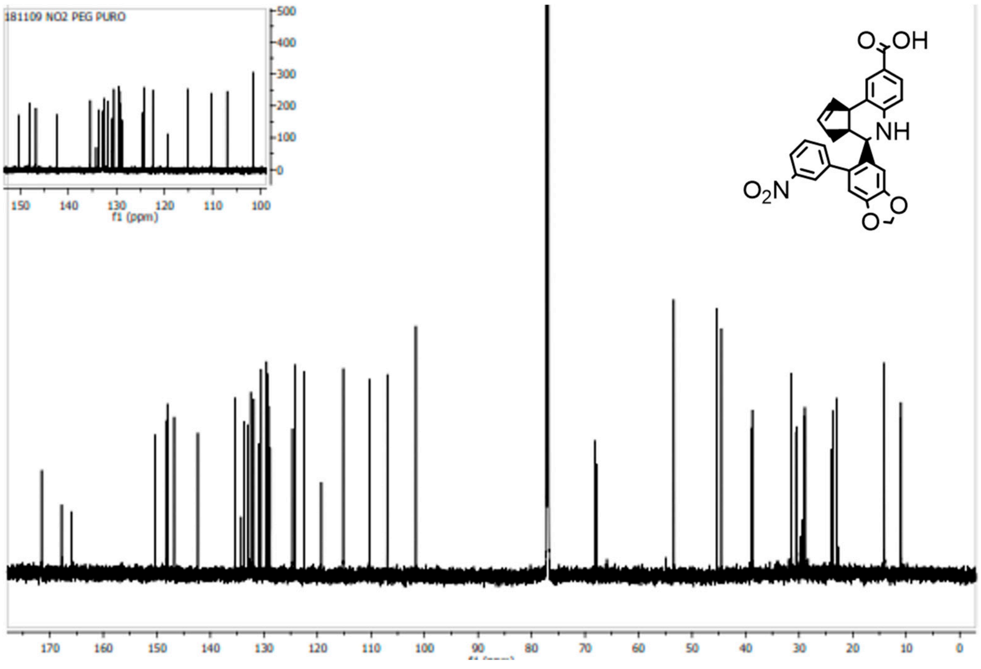

^{13}C NMR compound **4**.

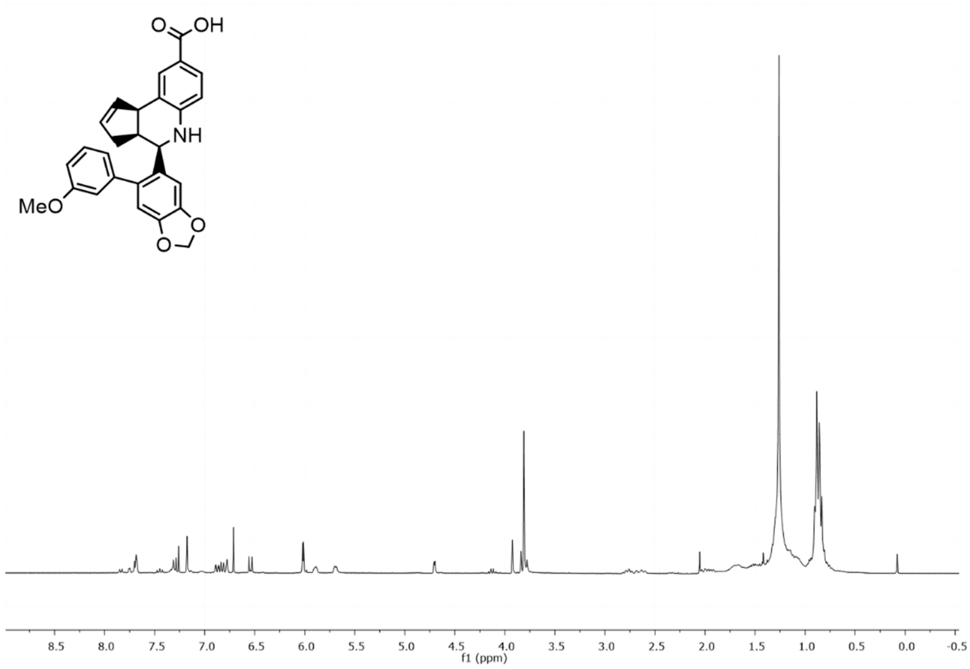

^{1}H NMR compound **5.**

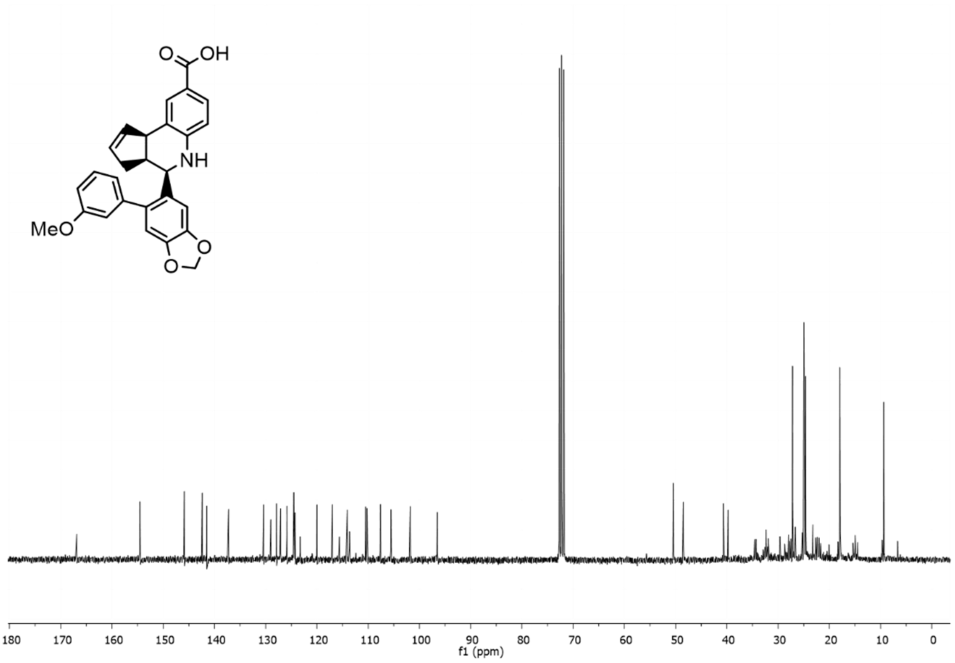

^{13}C NMR compound **5.**

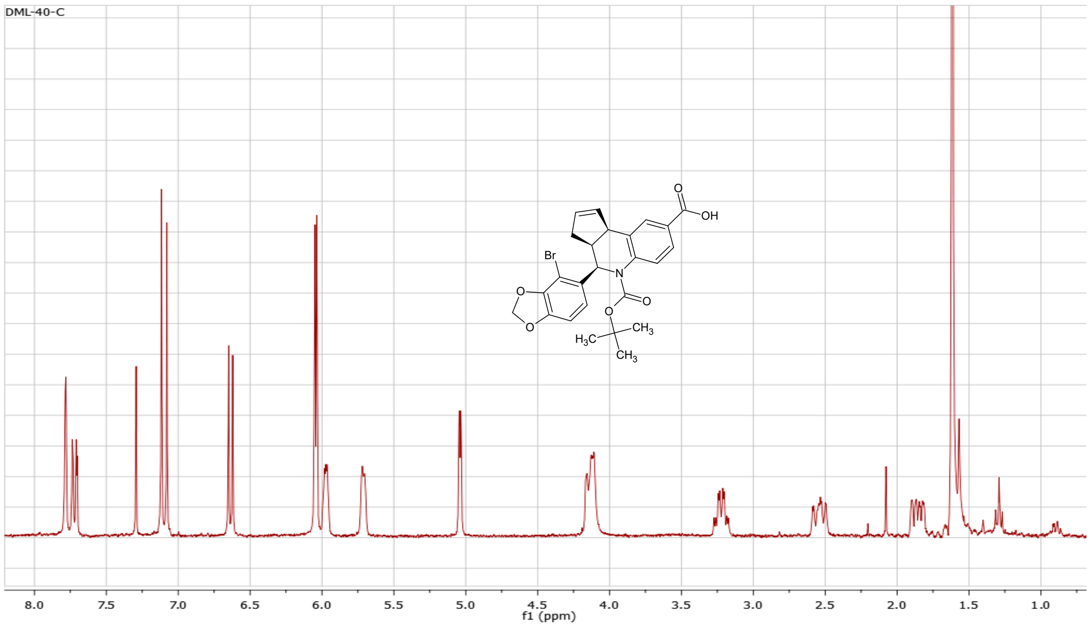

^{1}H NMR compound **7.**

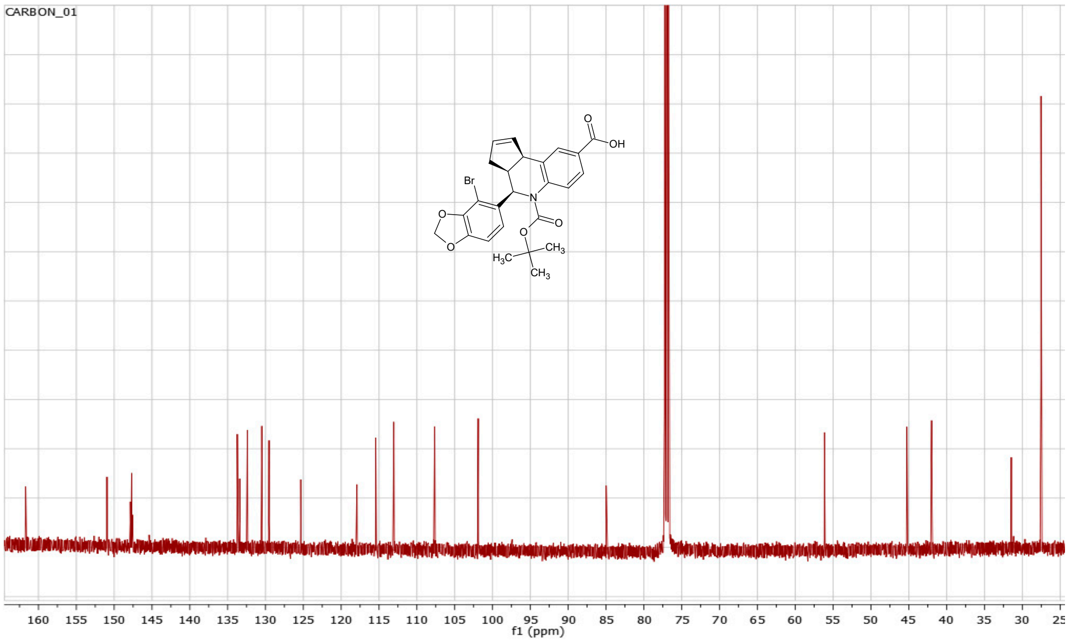

^{13}C NMR compound **7.**

HPLC data for purity statement.

HPLC conditions using an Agilent 1260	
COLUMN	CL-028 Zorbax SB C18, Agilent 4.6*150 mm, 5 µm.
SOLVENT	ACN:$H_2$0 60:40 v/v isocratic
WAVELENGTH	214 nm.
VOLUMEN	10 µL.
TEMPERATURE	25°C ±0.8°C
FLOW RATE	0.8 mL/min

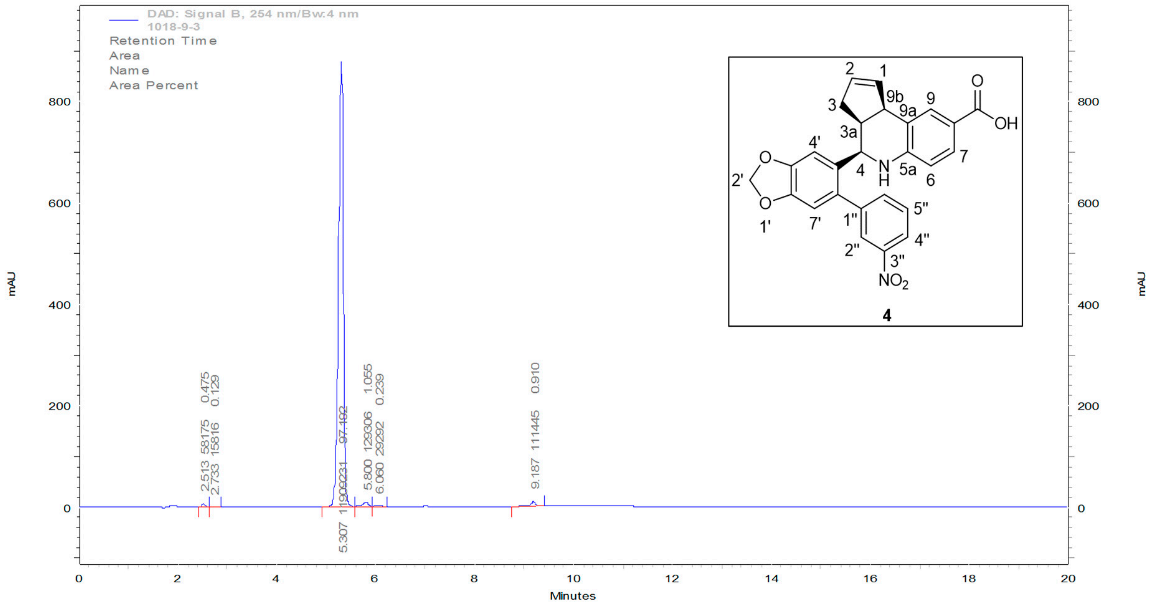

DAD: Signal B,
254 nm/Bw:4 nm
Results

Retention Time	Area	Area %	Height	Height %
2.513	58175	0.47	13598	0.72
2.733	15816	0.13	2955	0.16
5.307	11909231	97.19	1842132	96.95
5.800	129306	1.06	18581	0.98
6.060	29292	0.24	3695	0.19
9.187	111445	0.91	19194	1.01
Totals	12253265	100.00	1900155	100.00

Purity analysis determined by HPLC of the compound **4**. Percentage of purity= 97.19

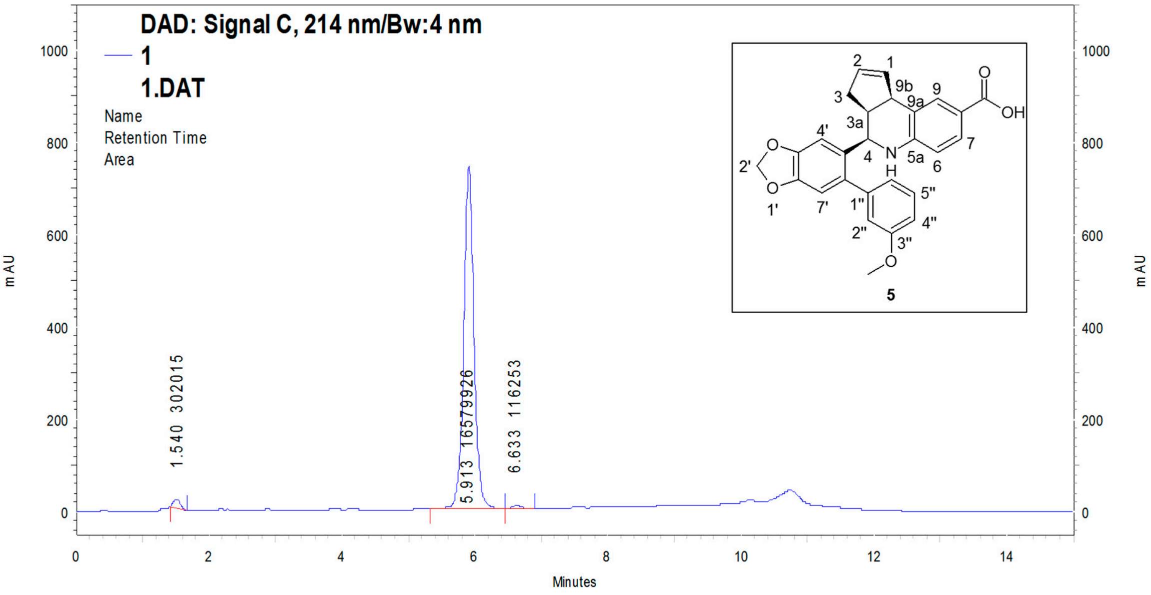

DAD: Signal C,
214 nm/Bw:4 nm
Results

Retention Time	Area	Area %	Height	Height %
1.540	302015	1.78	38645	2.40
5.913	16579926	97.54	1559492	96.71
6.633	116253	0.68	14366	0.89
Totals	16998194	100.00	1612503	100.00

Purity analysis determined by HPLC of the compound **5**. Percentage of purity= 97.59

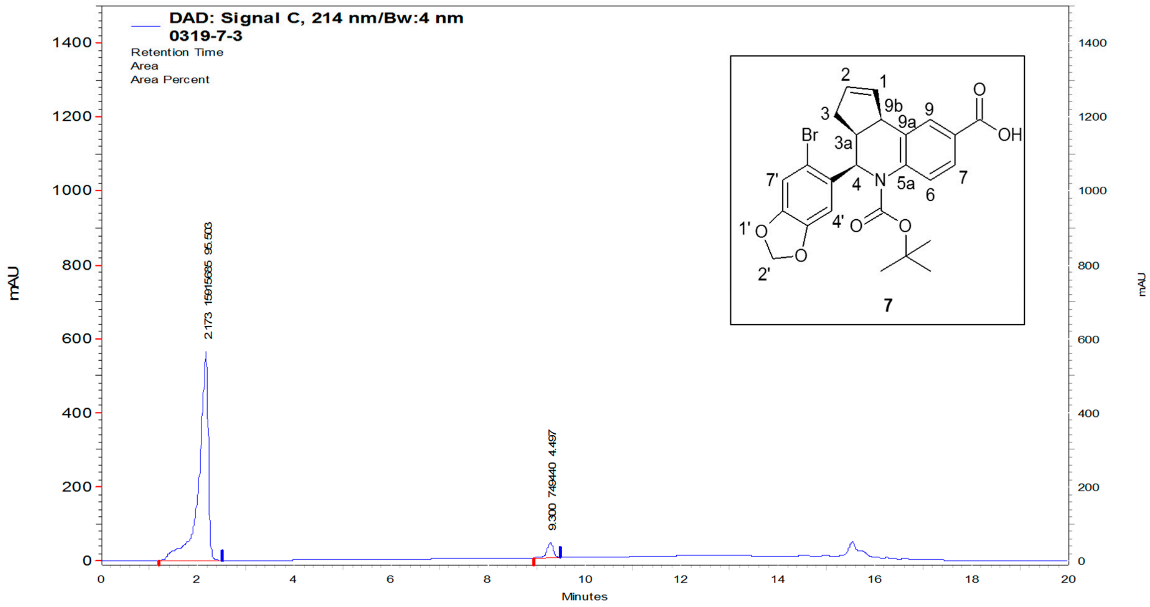

DAD: Signal C,
214 nm/Bw:4 nm
Results

Retention Time	Area	Area %	Height	Height %
2.173	15915685	95.50	1181777	93.26
9.300	749440	4.50	85356	6.74

Totals				
	16665125	100.00	1267133	100.00

Purity analysis determined by HPLC of the compound **7**. Percentage of purity= 95.50

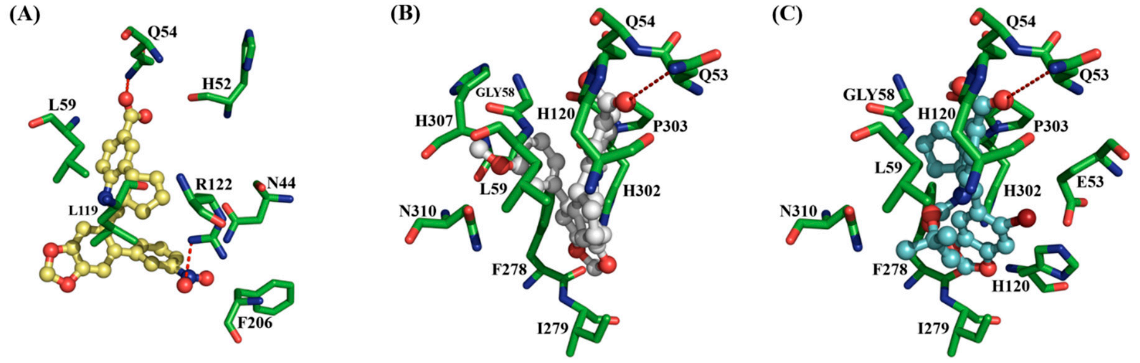

Figure S1. Binding pose yielded by docking analysis for A) Compound **4**, B) Compound **5** and C) Compound **7**. H bond interactions are depicted as red dashes.

FORCE FIELD PARAMETERS EMPLOYED IN MOLECULAR DYNAMICS SIMULATIONS

Force field parameters of compound4

```
!!index array str
"DRG"
!entry.DRG.unit.atoms table str name str type int typex int resx
int flags int seq int elmnt dbl chg
"OBG" "o" 0 1 131072 1 8 -0.216000
"NBF" "no" 0 1 131072 2 7 0.313200
"OBH" "o" 0 1 131072 3 8 -0.216000
"CBB" "ca" 0 1 131072 4 6 -0.177200
"CBA" "ca" 0 1 131072 5 6 -0.063000
"HBA" "ha" 0 1 131072 6 1 0.173000
"CBC" "ca" 0 1 131072 7 6 -0.067000
"HBC" "ha" 0 1 131072 8 1 0.169000
"CBD" "ca" 0 1 131072 9 6 -0.135000
"HBD" "ha" 0 1 131072 10 1 0.154000
"CBE" "ca" 0 1 131072 11 6 -0.069000
"HBE" "ha" 0 1 131072 12 1 0.153000
"CAZ" "cp" 0 1 131072 13 6 -0.036000
"CAE" "cp" 0 1 131072 14 6 -0.056000
"CAF" "ca" 0 1 131072 15 6 -0.108000
"HAF" "ha" 0 1 131072 16 1 0.155000
"CAD" "ca" 0 1 131072 17 6 0.047100
"OAI" "os" 0 1 131072 18 8 -0.337900
"CAH" "c3" 0 1 131072 19 6 0.321200
"HAH" "h2" 0 1 131072 20 1 0.067700
"HAI" "h2" 0 1 131072 21 1 0.067700
"OAG" "os" 0 1 131072 22 8 -0.331900
"CAB" "ca" 0 1 131072 23 6 0.055100
"CAA" "ca" 0 1 131072 24 6 -0.088000
"HAA" "ha" 0 1 131072 25 1 0.180000
"CAC" "ca" 0 1 131072 26 6 -0.051300
"CAJ" "c3" 0 1 131072 27 6 0.222800
"HAJ" "h1" 0 1 131072 28 1 0.042700
"CAK" "c3" 0 1 131072 29 6 -0.053700
"HAK" "hc" 0 1 131072 30 1 0.055700
"CAM" "c3" 0 1 131072 31 6 -0.050200
"HAM" "hc" 0 1 131072 32 1 0.042200
"HAN" "hc" 0 1 131072 33 1 0.042200
"CAV" "c2" 0 1 131072 34 6 -0.214200
"HAV" "ha" 0 1 131072 35 1 0.131000
"CAO" "c2" 0 1 131072 36 6 -0.139200
"HAO" "ha" 0 1 131072 37 1 0.155000
"CAL" "c3" 0 1 131072 38 6 0.020800
"HAL" "hc" 0 1 131072 39 1 0.065700
"CAN" "ca" 0 1 131072 40 6 -0.129300
"CAQ" "ca" 0 1 131072 41 6 -0.084000
"HAQ" "ha" 0 1 131072 42 1 0.154000
"CAU" "ca" 0 1 131072 43 6 -0.139600
```

```
"CAW" "c" 0 1 131072 44 6 0.910200
"OAY" "o" 0 1 131072 45 8 -0.829800
"OAX" "o" 0 1 131072 46 8 -0.829800
"CAT" "ca" 0 1 131072 47 6 -0.083000
"HAT" "ha" 0 1 131072 48 1 0.154000
"CAS" "ca" 0 1 131072 49 6 -0.197000
"HAS" "ha" 0 1 131072 50 1 0.107000
"CAP" "ca" 0 1 131072 51 6 0.072600
"NAR" "nh" 0 1 131072 52 7 -0.692600
"HAR" "hn" 0 1 131072 53 1 0.362800
!entry.DRG.unit.atomspertinfo table str pname str ptype int ptypex
int pelmnt dbl pchg
"OBG" "o" 0 -1 0.0
"NBF" "no" 0 -1 0.0
"OBH" "o" 0 -1 0.0
"CBB" "ca" 0 -1 0.0
"CBA" "ca" 0 -1 0.0
"HBA" "ha" 0 -1 0.0
"CBC" "ca" 0 -1 0.0
"HBC" "ha" 0 -1 0.0
"CBD" "ca" 0 -1 0.0
"HBD" "ha" 0 -1 0.0
"CBE" "ca" 0 -1 0.0
"HBE" "ha" 0 -1 0.0
"CAZ" "cp" 0 -1 0.0
"CAE" "cp" 0 -1 0.0
"CAF" "ca" 0 -1 0.0
"HAF" "ha" 0 -1 0.0
"CAD" "ca" 0 -1 0.0
"OAI" "os" 0 -1 0.0
"CAH" "c3" 0 -1 0.0
"HAH" "h2" 0 -1 0.0
"HAI" "h2" 0 -1 0.0
"OAG" "os" 0 -1 0.0
"CAB" "ca" 0 -1 0.0
"CAA" "ca" 0 -1 0.0
"HAA" "ha" 0 -1 0.0
"CAC" "ca" 0 -1 0.0
"CAJ" "c3" 0 -1 0.0
"HAJ" "h1" 0 -1 0.0
"CAK" "c3" 0 -1 0.0
"HAK" "hc" 0 -1 0.0
"CAM" "c3" 0 -1 0.0
"HAM" "hc" 0 -1 0.0
"HAN" "hc" 0 -1 0.0
"CAV" "c2" 0 -1 0.0
"HAV" "ha" 0 -1 0.0
"CAO" "c2" 0 -1 0.0
"HAO" "ha" 0 -1 0.0
"CAL" "c3" 0 -1 0.0
"HAL" "hc" 0 -1 0.0
"CAN" "ca" 0 -1 0.0
```

```
"CAQ" "ca" 0 -1 0.0
"HAQ" "ha" 0 -1 0.0
"CAU" "ca" 0 -1 0.0
"CAW" "c" 0 -1 0.0
"OAY" "o" 0 -1 0.0
"OAX" "o" 0 -1 0.0
"CAT" "ca" 0 -1 0.0
"HAT" "ha" 0 -1 0.0
"CAS" "ca" 0 -1 0.0
"HAS" "ha" 0 -1 0.0
"CAP" "ca" 0 -1 0.0
"NAR" "nh" 0 -1 0.0
"HAR" "hn" 0 -1 0.0
!entry.DRG.unit.boundbox array dbl
-1.000000
0.0
0.0
0.0
0.0
!entry.DRG.unit.childsequence single int
2
!entry.DRG.unit.connect array int
0
0
!entry.DRG.unit.connectivity table int atom1x int atom2x int flags
1 2 1
2 3 1
2 4 1
4 5 4
4 7 4
5 6 1
5 13 4
7 8 1
7 9 4
9 10 1
9 11 4
11 12 1
11 13 4
13 14 1
14 15 4
14 26 4
15 16 1
15 17 4
17 18 1
17 23 4
18 19 1
19 20 1
19 21 1
19 22 1
22 23 1
23 24 4
```

```
24 25 1
24 26 4
26 27 1
27 28 1
27 29 1
27 52 1
29 30 1
29 31 1
29 38 1
31 32 1
31 33 1
31 34 1
34 35 1
34 36 2
36 37 1
36 38 1
38 39 1
38 40 1
40 41 4
40 51 4
41 42 1
41 43 4
43 44 1
43 47 4
44 45 1
44 46 1
47 48 1
47 49 4
49 50 1
49 51 4
51 52 1
52 53 1
!entry.DRG.unit.hierarchy table str abovetype int abovex str
belowtype int belowx
"U" 0 "R" 1
"R" 1 "A" 1
"R" 1 "A" 2
"R" 1 "A" 3
"R" 1 "A" 4
"R" 1 "A" 5
"R" 1 "A" 6
"R" 1 "A" 7
"R" 1 "A" 8
"R" 1 "A" 9
"R" 1 "A" 10
"R" 1 "A" 11
"R" 1 "A" 12
"R" 1 "A" 13
"R" 1 "A" 14
"R" 1 "A" 15
"R" 1 "A" 16
"R" 1 "A" 17
```

```
"R" 1 "A" 18
"R" 1 "A" 19
"R" 1 "A" 20
"R" 1 "A" 21
"R" 1 "A" 22
"R" 1 "A" 23
"R" 1 "A" 24
"R" 1 "A" 25
"R" 1 "A" 26
"R" 1 "A" 27
"R" 1 "A" 28
"R" 1 "A" 29
"R" 1 "A" 30
"R" 1 "A" 31
"R" 1 "A" 32
"R" 1 "A" 33
"R" 1 "A" 34
"R" 1 "A" 35
"R" 1 "A" 36
"R" 1 "A" 37
"R" 1 "A" 38
"R" 1 "A" 39
"R" 1 "A" 40
"R" 1 "A" 41
"R" 1 "A" 42
"R" 1 "A" 43
"R" 1 "A" 44
"R" 1 "A" 45
"R" 1 "A" 46
"R" 1 "A" 47
"R" 1 "A" 48
"R" 1 "A" 49
"R" 1 "A" 50
"R" 1 "A" 51
"R" 1 "A" 52
"R" 1 "A" 53
!entry.DRG.unit.name single str
"DRG"
!entry.DRG.unit.positions table dbl x dbl y dbl z
1.760000 0.650000 -19.000000
0.930000 0.210000 -17.920000
-0.420000 0.670000 -17.870000
1.400000 -0.650000 -17.000000
0.580000 -1.080000 -15.930000
-0.440000 -0.720000 -15.850000
2.730000 -1.140000 -17.080000
3.380000 -0.820000 -17.890000
3.240000 -2.040000 -16.120000
4.260000 -2.420000 -16.200000
2.400000 -2.460000 -15.070000
2.790000 -3.170000 -14.330000
1.070000 -1.990000 -14.970000
```

```
0.230000 -2.480000 -13.960000
0.160000 -1.750000 -12.750000
0.800000 -0.880000 -12.590000
-0.710000 -2.140000 -11.790000
-0.800000 -1.690000 -10.520000
-2.060000 -2.230000 -10.080000
-1.982000 -2.450000 -9.015000
-2.880000 -1.562000 -10.342000
-2.170000 -3.480000 -10.790000
-1.520000 -3.220000 -11.960000
-1.500000 -3.960000 -13.080000
-2.160000 -4.830000 -13.170000
-0.620000 -3.610000 -14.130000
-0.480000 -4.470000 -15.230000
0.163000 -3.921000 -15.918000
-1.750000 -4.730000 -16.080000
-2.596000 -4.999000 -15.448000
-2.080000 -3.550000 -16.980000
-1.862000 -2.609000 -16.475000
-3.110000 -3.667000 -17.315000
-1.170000 -3.730000 -18.210000
-0.835000 -2.933000 -18.873000
-0.860000 -5.020000 -18.330000
-0.248000 -5.456000 -19.119000
-1.510000 -5.790000 -17.170000
-2.494000 -6.104000 -17.519000
-0.810000 -6.930000 -16.750000
-1.030000 -8.160000 -17.420000
-1.760000 -8.220000 -18.220000
-0.290000 -9.300000 -17.040000
-0.540000 -10.530000 -17.650000
0.400000 -11.200000 -18.120000
-1.710000 -10.950000 -17.780000
0.660000 -9.220000 -16.000000
1.240000 -10.100000 -15.710000
0.880000 -8.010000 -15.320000
1.620000 -7.950000 -14.510000
0.140000 -6.870000 -15.700000
0.310000 -5.700000 -14.930000
0.990000 -5.690000 -14.200000
!entry.DRG.unit.residueconnect table int c1x int c2x int c3x int
c4x int c5x int c6x
0 0 0 0 0 0
!entry.DRG.unit.residues table str name int seq int childseq int
startatomx str restype int imagingx
"DRG" 1 54 1 "?" 0
!entry.DRG.unit.residuesPdbSequenceNumber array int
0
!entry.DRG.unit.solventcap array dbl
-1.000000
0.0
0.0
```

```
0.0
0.0
!entry.DRG.unit.velocities table dbl x dbl y dbl z
0.0 0.0 0.0
0.0 0.0 0.0
0.0 0.0 0.0
0.0 0.0 0.0
0.0 0.0 0.0
0.0 0.0 0.0
0.0 0.0 0.0
0.0 0.0 0.0
0.0 0.0 0.0
0.0 0.0 0.0
0.0 0.0 0.0
0.0 0.0 0.0
0.0 0.0 0.0
0.0 0.0 0.0
0.0 0.0 0.0
0.0 0.0 0.0
0.0 0.0 0.0
0.0 0.0 0.0
0.0 0.0 0.0
0.0 0.0 0.0
0.0 0.0 0.0
0.0 0.0 0.0
0.0 0.0 0.0
0.0 0.0 0.0
0.0 0.0 0.0
0.0 0.0 0.0
0.0 0.0 0.0
0.0 0.0 0.0
0.0 0.0 0.0
0.0 0.0 0.0
0.0 0.0 0.0
0.0 0.0 0.0
0.0 0.0 0.0
0.0 0.0 0.0
0.0 0.0 0.0
0.0 0.0 0.0
0.0 0.0 0.0
0.0 0.0 0.0
0.0 0.0 0.0
0.0 0.0 0.0
0.0 0.0 0.0
0.0 0.0 0.0
0.0 0.0 0.0
0.0 0.0 0.0
0.0 0.0 0.0
0.0 0.0 0.0
0.0 0.0 0.0
0.0 0.0 0.0
0.0 0.0 0.0
```

```
0.0 0.0 0.0
0.0 0.0 0.0
0.0 0.0 0.0
0.0 0.0 0.0
remark goes here
MASS
BOND
ANGLE
DIHE
IMPROPER
ca-ca-ca-no 1.1 180.0 2.0 Using default value
ca-cp-ca-ha 1.1 180.0 2.0 General improper torsional angle (2
general atom types)
ca-ca-ca-ha 1.1 180.0 2.0 General improper torsional angle (2
general atom types)
ca-ca-cp-cp 1.1 180.0 2.0 Using default value
ca-ca-ca-os 1.1 180.0 2.0 Using default value
c3-ca-ca-cp 1.1 180.0 2.0 Using default value
c2-c3-c2-ha 1.1 180.0 2.0 Using default value
c -ca-ca-ca 1.1 180.0 2.0 Using default value
ca-o -c -o 1.1 180.0 2.0 General improper torsional angle (1
general atom type)
ca-ca-ca-nh 1.1 180.0 2.0 Using default value
c3-ca-nh-hn 1.1 180.0 2.0 Using default value
NONBON
```

Force field parameters of compound5

```
!!index array str
"DRG"
!entry.DRG.unit.atoms table str name str type int typex int resx
int flags int seq int elmnt dbl chg
"CAK" "c3" 0 1 131072 1 6 -0.096700
"HAK" "hc" 0 1 131072 2 1 0.054700
"CAM" "c3" 0 1 131072 3 6 -0.045200
"HAM" "hc" 0 1 131072 4 1 0.041200
"HAN" "hc" 0 1 131072 5 1 0.041200
"CAV" "c2" 0 1 131072 6 6 -0.216200
"HAV" "ha" 0 1 131072 7 1 0.127000
"CAO" "c2" 0 1 131072 8 6 -0.136200
"HAO" "ha" 0 1 131072 9 1 0.154000
"CAL" "c3" 0 1 131072 10 6 0.015800
"HAL" "hc" 0 1 131072 11 1 0.061700
"CAN" "ca" 0 1 131072 12 6 -0.134300
"CAQ" "ca" 0 1 131072 13 6 -0.095000
"HAQ" "ha" 0 1 131072 14 1 0.151000
"CAU" "ca" 0 1 131072 15 6 -0.128600
"CAW" "c" 0 1 131072 16 6 0.908200
"OAY" "o" 0 1 131072 17 8 -0.831300
"OAX" "o" 0 1 131072 18 8 -0.831300
"CAT" "ca" 0 1 131072 19 6 -0.101000
"HAT" "ha" 0 1 131072 20 1 0.153000
"CAS" "ca" 0 1 131072 21 6 -0.151000
"HAS" "ha" 0 1 131072 22 1 0.118000
"CAP" "ca" 0 1 131072 23 6 0.052600
"NAR" "nh" 0 1 131072 24 7 -0.686600
"HAR" "hn" 0 1 131072 25 1 0.362800
"CAJ" "c3" 0 1 131072 26 6 0.221800
"HAJ" "h1" 0 1 131072 27 1 0.076700
"CAC" "ca" 0 1 131072 28 6 -0.030300
"CAA" "ca" 0 1 131072 29 6 -0.107000
"HAA" "ha" 0 1 131072 30 1 0.162000
"CAB" "ca" 0 1 131072 31 6 0.053100
"OAG" "os" 0 1 131072 32 8 -0.336900
"CAH" "c3" 0 1 131072 33 6 0.322200
"HAH" "h2" 0 1 131072 34 1 0.064200
"HAI" "h2" 0 1 131072 35 1 0.064200
"OAI" "os" 0 1 131072 36 8 -0.339900
"CAD" "ca" 0 1 131072 37 6 0.040100
"CAF" "ca" 0 1 131072 38 6 -0.103000
"HAF" "ha" 0 1 131072 39 1 0.155000
"CAE" "cp" 0 1 131072 40 6 -0.041000
"CAZ" "cp" 0 1 131072 41 6 -0.012000
"CBA" "ca" 0 1 131072 42 6 -0.142000
"HBA" "ha" 0 1 131072 43 1 0.154000
"CBE" "ca" 0 1 131072 44 6 -0.149000
"HBE" "ha" 0 1 131072 45 1 0.148000
"CBD" "ca" 0 1 131072 46 6 -0.097000
```

"HBD" "ha" 0 1 131072 47 1 0.135000
"CBC" "ca" 0 1 131072 48 6 -0.194000
"HBC" "ha" 0 1 131072 49 1 0.136000
"CBB" "ca" 0 1 131072 50 6 0.118100
"OBF" "os" 0 1 131072 51 8 -0.331900
"CBG" "c3" 0 1 131072 52 6 0.114700
"HBH" "h1" 0 1 131072 53 1 0.042700
"HBI" "h1" 0 1 131072 54 1 0.042700
"HBG" "h1" 0 1 131072 55 1 0.042700
!entry.DRG.unit.atomspertinfo table str pname str ptype int ptypex int pelmnt dbl pchg
"CAK" "c3" 0 -1 0.0
"HAK" "hc" 0 -1 0.0
"CAM" "c3" 0 -1 0.0
"HAM" "hc" 0 -1 0.0
"HAN" "hc" 0 -1 0.0
"CAV" "c2" 0 -1 0.0
"HAV" "ha" 0 -1 0.0
"CAO" "c2" 0 -1 0.0
"HAO" "ha" 0 -1 0.0
"CAL" "c3" 0 -1 0.0
"HAL" "hc" 0 -1 0.0
"CAN" "ca" 0 -1 0.0
"CAQ" "ca" 0 -1 0.0
"HAQ" "ha" 0 -1 0.0
"CAU" "ca" 0 -1 0.0
"CAW" "c" 0 -1 0.0
"OAY" "o" 0 -1 0.0
"OAX" "o" 0 -1 0.0
"CAT" "ca" 0 -1 0.0
"HAT" "ha" 0 -1 0.0
"CAS" "ca" 0 -1 0.0
"HAS" "ha" 0 -1 0.0
"CAP" "ca" 0 -1 0.0
"NAR" "nh" 0 -1 0.0
"HAR" "hn" 0 -1 0.0
"CAJ" "c3" 0 -1 0.0
"HAJ" "h1" 0 -1 0.0
"CAC" "ca" 0 -1 0.0
"CAA" "ca" 0 -1 0.0
"HAA" "ha" 0 -1 0.0
"CAB" "ca" 0 -1 0.0
"OAG" "os" 0 -1 0.0
"CAH" "c3" 0 -1 0.0
"HAH" "h2" 0 -1 0.0
"HAI" "h2" 0 -1 0.0
"OAI" "os" 0 -1 0.0
"CAD" "ca" 0 -1 0.0
"CAF" "ca" 0 -1 0.0
"HAF" "ha" 0 -1 0.0
"CAE" "cp" 0 -1 0.0
"CAZ" "cp" 0 -1 0.0

```
"CBA" "ca" 0 -1 0.0
"HBA" "ha" 0 -1 0.0
"CBE" "ca" 0 -1 0.0
"HBE" "ha" 0 -1 0.0
"CBD" "ca" 0 -1 0.0
"HBD" "ha" 0 -1 0.0
"CBC" "ca" 0 -1 0.0
"HBC" "ha" 0 -1 0.0
"CBB" "ca" 0 -1 0.0
"OBF" "os" 0 -1 0.0
"CBG" "c3" 0 -1 0.0
"HBH" "h1" 0 -1 0.0
"HBI" "h1" 0 -1 0.0
"HBG" "h1" 0 -1 0.0
!entry.DRG.unit.boundbox array dbl
-1.000000
0.0
0.0
0.0
0.0
!entry.DRG.unit.childsequence single int
2
!entry.DRG.unit.connect array int
0
0
!entry.DRG.unit.connectivity table int atom1x int atom2x int flags
1 2 1
1 3 1
1 10 1
1 26 1
3 4 1
3 5 1
3 6 1
6 7 1
6 8 2
8 9 1
8 10 1
10 11 1
10 12 1
12 13 4
12 23 4
13 14 1
13 15 4
15 16 1
15 19 4
16 17 1
16 18 1
19 20 1
19 21 4
21 22 1
21 23 4
```

```
23 24 1
24 25 1
24 26 1
26 27 1
26 28 1
28 29 4
28 40 4
29 30 1
29 31 4
31 32 1
31 37 4
32 33 1
33 34 1
33 35 1
33 36 1
36 37 1
37 38 4
38 39 1
38 40 4
40 41 1
41 42 4
41 44 4
42 43 1
42 50 4
44 45 1
44 46 4
46 47 1
46 48 4
48 49 1
48 50 4
50 51 1
51 52 1
52 53 1
52 54 1
52 55 1
!entry.DRG.unit.hierarchy table str abovetype int abovex str
belowtype int belowx
"U" 0 "R" 1
"R" 1 "A" 1
"R" 1 "A" 2
"R" 1 "A" 3
"R" 1 "A" 4
"R" 1 "A" 5
"R" 1 "A" 6
"R" 1 "A" 7
"R" 1 "A" 8
"R" 1 "A" 9
"R" 1 "A" 10
"R" 1 "A" 11
"R" 1 "A" 12
"R" 1 "A" 13
"R" 1 "A" 14
```

```
"R" 1 "A" 15
"R" 1 "A" 16
"R" 1 "A" 17
"R" 1 "A" 18
"R" 1 "A" 19
"R" 1 "A" 20
"R" 1 "A" 21
"R" 1 "A" 22
"R" 1 "A" 23
"R" 1 "A" 24
"R" 1 "A" 25
"R" 1 "A" 26
"R" 1 "A" 27
"R" 1 "A" 28
"R" 1 "A" 29
"R" 1 "A" 30
"R" 1 "A" 31
"R" 1 "A" 32
"R" 1 "A" 33
"R" 1 "A" 34
"R" 1 "A" 35
"R" 1 "A" 36
"R" 1 "A" 37
"R" 1 "A" 38
"R" 1 "A" 39
"R" 1 "A" 40
"R" 1 "A" 41
"R" 1 "A" 42
"R" 1 "A" 43
"R" 1 "A" 44
"R" 1 "A" 45
"R" 1 "A" 46
"R" 1 "A" 47
"R" 1 "A" 48
"R" 1 "A" 49
"R" 1 "A" 50
"R" 1 "A" 51
"R" 1 "A" 52
"R" 1 "A" 53
"R" 1 "A" 54
"R" 1 "A" 55
!entry.DRG.unit.name single str
"DRG"
!entry.DRG.unit.positions table dbl x dbl y dbl z
1.575000 -7.281000 -15.365000
1.740000 -7.762000 -14.401000
1.650000 -5.749000 -15.191000
2.526000 -5.348000 -15.701000
1.584000 -5.553000 -14.121000
0.393000 -5.225000 -15.814000
0.174000 -4.166000 -15.947000
-0.431000 -6.216000 -16.189000
```

```
-1.389000 -6.060000 -16.685000
0.130000 -7.572000 -15.841000
-0.421000 -7.941000 -14.976000
-0.015000 -8.576000 -16.930000
-1.283000 -8.850000 -17.438000
-2.142000 -8.291000 -17.067000
-1.470000 -9.831000 -18.416000
-2.795000 -10.123000 -18.959000
-3.370000 -11.283000 -18.517000
-3.466000 -9.463000 -19.764000
-0.368000 -10.560000 -18.889000
-0.512000 -11.333000 -19.644000
0.901000 -10.301000 -18.401000
1.752000 -10.868000 -18.779000
1.104000 -9.304000 -17.413000
2.371000 -9.091000 -16.872000
3.055000 -9.820000 -16.835000
2.651000 -7.754000 -16.363000
2.657000 -7.055000 -17.199000
4.015000 -7.711000 -15.708000
4.835000 -6.590000 -15.976000
4.493000 -5.771000 -16.609000
6.085000 -6.581000 -15.400000
7.082000 -5.616000 -15.494000
8.197000 -6.090000 -14.704000
8.412000 -5.381000 -13.904000
9.022000 -6.270000 -15.393000
7.830000 -7.371000 -14.140000
6.538000 -7.644000 -14.576000
5.749000 -8.739000 -14.305000
6.103000 -9.555000 -13.675000
4.455000 -8.755000 -14.883000
3.616000 -9.919000 -14.569000
2.521000 -9.763000 -13.706000
2.310000 -8.794000 -13.253000
3.876000 -11.164000 -15.150000
4.725000 -11.284000 -15.822000
3.049000 -12.254000 -14.870000
3.259000 -13.221000 -15.327000
1.963000 -12.120000 -14.016000
1.321000 -12.974000 -13.799000
1.703000 -10.865000 -13.434000
0.603000 -10.831000 -12.600000
0.306000 -12.033000 -11.897000
1.152000 -12.303000 -11.265000
0.130000 -12.778000 -12.673000
-0.577000 -11.883000 -11.276000
!entry.DRG.unit.residueconnect table int c1x int c2x int c3x int
c4x int c5x int c6x
0 0 0 0 0 0
!entry.DRG.unit.residues table str name int seq int childseq int
startatomx str restype int imagingx
```

```
"DRG" 1 56 1 "?" 0
!entry.DRG.unit.residuesPdbSequenceNumber array int
0
!entry.DRG.unit.solventcap array dbl
-1.000000
0.0
0.0
0.0
0.0
!entry.DRG.unit.velocities table dbl x dbl y dbl z
0.0 0.0 0.0
0.0 0.0 0.0
0.0 0.0 0.0
0.0 0.0 0.0
0.0 0.0 0.0
0.0 0.0 0.0
0.0 0.0 0.0
0.0 0.0 0.0
0.0 0.0 0.0
0.0 0.0 0.0
0.0 0.0 0.0
0.0 0.0 0.0
0.0 0.0 0.0
0.0 0.0 0.0
0.0 0.0 0.0
0.0 0.0 0.0
0.0 0.0 0.0
0.0 0.0 0.0
0.0 0.0 0.0
0.0 0.0 0.0
0.0 0.0 0.0
0.0 0.0 0.0
0.0 0.0 0.0
0.0 0.0 0.0
0.0 0.0 0.0
0.0 0.0 0.0
0.0 0.0 0.0
0.0 0.0 0.0
0.0 0.0 0.0
0.0 0.0 0.0
0.0 0.0 0.0
0.0 0.0 0.0
0.0 0.0 0.0
0.0 0.0 0.0
0.0 0.0 0.0
0.0 0.0 0.0
0.0 0.0 0.0
0.0 0.0 0.0
0.0 0.0 0.0
0.0 0.0 0.0
0.0 0.0 0.0
0.0 0.0 0.0
```

```
0.0 0.0 0.0
0.0 0.0 0.0
0.0 0.0 0.0
0.0 0.0 0.0
0.0 0.0 0.0
0.0 0.0 0.0
0.0 0.0 0.0
0.0 0.0 0.0
0.0 0.0 0.0
0.0 0.0 0.0
0.0 0.0 0.0
0.0 0.0 0.0
0.0 0.0 0.0
remark goes here
MASS
BOND
ANGLE
DIHE
IMPROPER
c2-c3-c2-ha 1.1 180.0 2.0 Using default value
ca-ca-ca-ha 1.1 180.0 2.0 General improper torsional angle (2
general atom types)
c -ca-ca-ca 1.1 180.0 2.0 Using default value
ca-o -c -o 1.1 180.0 2.0 General improper torsional angle (1
general atom type)
ca-ca-ca-nh 1.1 180.0 2.0 Using default value
c3-ca-nh-hn 1.1 180.0 2.0 Using default value
c3-ca-ca-cp 1.1 180.0 2.0 Using default value
ca-ca-ca-os 1.1 180.0 2.0 Using default value
ca-cp-ca-ha 1.1 180.0 2.0 General improper torsional angle (2
general atom types)
ca-ca-cp-cp 1.1 180.0 2.0 Using default value
NONBON
```

Force field parameters of compound7
!!index array str
"DRG"
!entry.DRG.unit.atoms table str name str type int typex int resx int flags int seq int elmnt dbl chg
"BRAJ" "br" 0 1 131072 1 5 -0.071100
"CAE" "ca" 0 1 131072 2 6 -0.015900
"CAF" "ca" 0 1 131072 3 6 -0.107000
"HAF" "ha" 0 1 131072 4 1 0.161000
"CAD" "ca" 0 1 131072 5 6 0.052100
"OAI" "os" 0 1 131072 6 8 -0.338900
"CAH" "c3" 0 1 131072 7 6 0.321200
"HAH" "h2" 0 1 131072 8 1 0.065700
"HAI" "h2" 0 1 131072 9 1 0.065700
"OAG" "os" 0 1 131072 10 8 -0.331900
"CAB" "ca" 0 1 131072 11 6 0.050100
"CAA" "ca" 0 1 131072 12 6 -0.064000
"HAA" "ha" 0 1 131072 13 1 0.185000
"CAC" "ca" 0 1 131072 14 6 -0.038300
"CAK" "c3" 0 1 131072 15 6 0.138000
"HAK" "h1" 0 1 131072 16 1 0.095700
"CAL" "c3" 0 1 131072 17 6 -0.084700
"HAL" "hc" 0 1 131072 18 1 0.053700
"CAN" "c3" 0 1 131072 19 6 -0.057200
"HAN" "hc" 0 1 131072 20 1 0.054700
"HAO" "hc" 0 1 131072 21 1 0.054700
"CAY" "c2" 0 1 131072 22 6 -0.201200
"HAY" "ha" 0 1 131072 23 1 0.130000
"CAP" "c2" 0 1 131072 24 6 -0.145200
"HAP" "ha" 0 1 131072 25 1 0.151000
"CAM" "c3" 0 1 131072 26 6 0.011800
"HAM" "hc" 0 1 131072 27 1 0.064700
"CAO" "ca" 0 1 131072 28 6 -0.124300
"CAR" "ca" 0 1 131072 29 6 -0.098000
"HAR" "ha" 0 1 131072 30 1 0.156000
"CAX" "ca" 0 1 131072 31 6 -0.119600
"CBE" "c" 0 1 131072 32 6 0.908200
"OBG" "o" 0 1 131072 33 8 -0.827300
"OBF" "o" 0 1 131072 34 8 -0.827300
"CAW" "ca" 0 1 131072 35 6 -0.103000
"HAW" "ha" 0 1 131072 36 1 0.157000
"CAT" "ca" 0 1 131072 37 6 -0.128000
"HAT" "ha" 0 1 131072 38 1 0.131000
"CAQ" "ca" 0 1 131072 39 6 -0.004400
"NAS" "n" 0 1 131072 40 7 -0.305000
"CAU" "c" 0 1 131072 41 6 0.744100
"OAV" "o" 0 1 131072 42 8 -0.625000
"OAZ" "os" 0 1 131072 43 8 -0.417900
"CBA" "c3" 0 1 131072 44 6 0.182800
"CBC" "c3" 0 1 131072 45 6 -0.111767
"HBF" "hc" 0 1 131072 46 1 0.048700

"HBG" "hc" 0 1 131072 47 1 0.048700
"HBE" "hc" 0 1 131072 48 1 0.048700
"CBD" "c3" 0 1 131072 49 6 -0.111767
"HBI" "hc" 0 1 131072 50 1 0.048700
"HBJ" "hc" 0 1 131072 51 1 0.048700
"HBH" "hc" 0 1 131072 52 1 0.048700
"CBB" "c3" 0 1 131072 53 6 -0.111767
"HBC" "hc" 0 1 131072 54 1 0.048700
"HBD" "hc" 0 1 131072 55 1 0.048700
"HBB" "hc" 0 1 131072 56 1 0.048700
!entry.DRG.unit.atomspertinfo table str pname str ptype int ptypex int pelmnt dbl pchg
"BRAJ" "br" 0 -1 0.0
"CAE" "ca" 0 -1 0.0
"CAF" "ca" 0 -1 0.0
"HAF" "ha" 0 -1 0.0
"CAD" "ca" 0 -1 0.0
"OAI" "os" 0 -1 0.0
"CAH" "c3" 0 -1 0.0
"HAH" "h2" 0 -1 0.0
"HAI" "h2" 0 -1 0.0
"OAG" "os" 0 -1 0.0
"CAB" "ca" 0 -1 0.0
"CAA" "ca" 0 -1 0.0
"HAA" "ha" 0 -1 0.0
"CAC" "ca" 0 -1 0.0
"CAK" "c3" 0 -1 0.0
"HAK" "h1" 0 -1 0.0
"CAL" "c3" 0 -1 0.0
"HAL" "hc" 0 -1 0.0
"CAN" "c3" 0 -1 0.0
"HAN" "hc" 0 -1 0.0
"HAO" "hc" 0 -1 0.0
"CAY" "c2" 0 -1 0.0
"HAY" "ha" 0 -1 0.0
"CAP" "c2" 0 -1 0.0
"HAP" "ha" 0 -1 0.0
"CAM" "c3" 0 -1 0.0
"HAM" "hc" 0 -1 0.0
"CAO" "ca" 0 -1 0.0
"CAR" "ca" 0 -1 0.0
"HAR" "ha" 0 -1 0.0
"CAX" "ca" 0 -1 0.0
"CBE" "c" 0 -1 0.0
"OBG" "o" 0 -1 0.0
"OBF" "o" 0 -1 0.0
"CAW" "ca" 0 -1 0.0
"HAW" "ha" 0 -1 0.0
"CAT" "ca" 0 -1 0.0
"HAT" "ha" 0 -1 0.0
"CAQ" "ca" 0 -1 0.0
"NAS" "n" 0 -1 0.0

```
"CAU" "c" 0 -1 0.0
"OAV" "o" 0 -1 0.0
"OAZ" "os" 0 -1 0.0
"CBA" "c3" 0 -1 0.0
"CBC" "c3" 0 -1 0.0
"HBF" "hc" 0 -1 0.0
"HBG" "hc" 0 -1 0.0
"HBE" "hc" 0 -1 0.0
"CBD" "c3" 0 -1 0.0
"HBI" "hc" 0 -1 0.0
"HBJ" "hc" 0 -1 0.0
"HBH" "hc" 0 -1 0.0
"CBB" "c3" 0 -1 0.0
"HBC" "hc" 0 -1 0.0
"HBD" "hc" 0 -1 0.0
"HBB" "hc" 0 -1 0.0
!entry.DRG.unit.boundbox array dbl
-1.000000
0.0
0.0
0.0
0.0
!entry.DRG.unit.childsequence single int
2
!entry.DRG.unit.connect array int
0
0
!entry.DRG.unit.connectivity table int atom1x int atom2x int flags
1 2 1
2 3 4
2 14 4
3 4 1
3 5 4
5 6 1
5 11 4
6 7 1
7 8 1
7 9 1
7 10 1
10 11 1
11 12 4
12 13 1
12 14 4
14 15 1
15 16 1
15 17 1
15 40 1
17 18 1
17 19 1
17 26 1
19 20 1
```

```
19 21 1
19 22 1
22 23 1
22 24 2
24 25 1
24 26 1
26 27 1
26 28 1
28 29 4
28 39 4
29 30 1
29 31 4
31 32 1
31 35 4
32 33 1
32 34 1
35 36 1
35 37 4
37 38 1
37 39 4
39 40 1
40 41 1
41 42 2
41 43 1
43 44 1
44 45 1
44 49 1
44 53 1
45 46 1
45 47 1
45 48 1
49 50 1
49 51 1
49 52 1
53 54 1
53 55 1
53 56 1
!entry.DRG.unit.hierarchy table str abovetype int abovex str
belowtype int belowx
"U" 0 "R" 1
"R" 1 "A" 1
"R" 1 "A" 2
"R" 1 "A" 3
"R" 1 "A" 4
"R" 1 "A" 5
"R" 1 "A" 6
"R" 1 "A" 7
"R" 1 "A" 8
"R" 1 "A" 9
"R" 1 "A" 10
"R" 1 "A" 11
"R" 1 "A" 12
```

```
"R" 1 "A" 13
"R" 1 "A" 14
"R" 1 "A" 15
"R" 1 "A" 16
"R" 1 "A" 17
"R" 1 "A" 18
"R" 1 "A" 19
"R" 1 "A" 20
"R" 1 "A" 21
"R" 1 "A" 22
"R" 1 "A" 23
"R" 1 "A" 24
"R" 1 "A" 25
"R" 1 "A" 26
"R" 1 "A" 27
"R" 1 "A" 28
"R" 1 "A" 29
"R" 1 "A" 30
"R" 1 "A" 31
"R" 1 "A" 32
"R" 1 "A" 33
"R" 1 "A" 34
"R" 1 "A" 35
"R" 1 "A" 36
"R" 1 "A" 37
"R" 1 "A" 38
"R" 1 "A" 39
"R" 1 "A" 40
"R" 1 "A" 41
"R" 1 "A" 42
"R" 1 "A" 43
"R" 1 "A" 44
"R" 1 "A" 45
"R" 1 "A" 46
"R" 1 "A" 47
"R" 1 "A" 48
"R" 1 "A" 49
"R" 1 "A" 50
"R" 1 "A" 51
"R" 1 "A" 52
"R" 1 "A" 53
"R" 1 "A" 54
"R" 1 "A" 55
"R" 1 "A" 56
!entry.DRG.unit.name single str
"DRG"
!entry.DRG.unit.positions table dbl x dbl y dbl z
3.780000 -6.130000 -18.464000
4.840000 -6.666000 -17.003000
6.020000 -5.917000 -16.811000
6.289000 -5.093000 -17.472000
6.814000 -6.280000 -15.746000
```

8.018000 -5.727000 -15.324000
8.423000 -6.465000 -14.146000
9.393000 -6.932000 -14.317000
8.388000 -5.770000 -13.307000
7.425000 -7.482000 -13.894000
6.454000 -7.343000 -14.880000
5.299000 -8.072000 -15.061000
5.026000 -8.879000 -14.381000
4.483000 -7.733000 -16.161000
3.247000 -8.539000 -16.475000
3.095000 -8.374000 -17.542000
3.416000 -10.060000 -16.369000
4.287000 -10.344000 -16.960000
3.572000 -10.679000 -14.970000
3.261000 -9.972000 -14.201000
4.597000 -11.044000 -14.902000
2.676000 -11.879000 -14.975000
2.650000 -12.624000 -14.180000
1.909000 -11.927000 -16.075000
1.184000 -12.714000 -16.280000
2.172000 -10.758000 -16.994000
2.440000 -11.130000 -17.983000
0.950000 -9.902000 -17.098000
-0.148000 -10.400000 -17.804000
-0.079000 -11.372000 -18.292000
-1.335000 -9.666000 -17.892000
-2.457000 -10.234000 -18.643000
-3.016000 -11.358000 -18.102000
-2.963000 -9.830000 -19.698000
-1.434000 -8.420000 -17.263000
-2.365000 -7.855000 -17.318000
-0.348000 -7.901000 -16.568000
-0.429000 -6.927000 -16.085000
0.856000 -8.627000 -16.484000
2.006000 -8.061000 -15.859000
1.953000 -7.691000 -14.506000
2.901000 -7.660000 -13.706000
0.705000 -7.288000 -14.103000
0.464000 -5.880000 -13.823000
0.194000 -5.888000 -12.318000
0.031000 -4.867000 -11.973000
1.094000 -6.327000 -11.887000
-0.693000 -6.487000 -12.111000
1.697000 -5.050000 -14.134000
2.560000 -5.475000 -13.620000
1.451000 -4.054000 -13.765000
1.876000 -5.054000 -15.209000
-0.758000 -5.381000 -14.576000
-0.571000 -5.435000 -15.648000
-0.883000 -4.353000 -14.235000
-1.618000 -6.002000 -14.326000

!entry.DRG.unit.residueconnect table int c1x int c2x int c3x int
c4x int c5x int c6x
0 0 0 0 0 0
!entry.DRG.unit.residues table str name int seq int childseq int
startatomx str restype int imagingx
"DRG" 1 57 1 "?" 0
!entry.DRG.unit.residuesPdbSequenceNumber array int
0
!entry.DRG.unit.solventcap array dbl
-1.000000
0.0
0.0
0.0
0.0
!entry.DRG.unit.velocities table dbl x dbl y dbl z
0.0 0.0 0.0
0.0 0.0 0.0
0.0 0.0 0.0
0.0 0.0 0.0
0.0 0.0 0.0
0.0 0.0 0.0
0.0 0.0 0.0
0.0 0.0 0.0
0.0 0.0 0.0
0.0 0.0 0.0
0.0 0.0 0.0
0.0 0.0 0.0
0.0 0.0 0.0
0.0 0.0 0.0
0.0 0.0 0.0
0.0 0.0 0.0
0.0 0.0 0.0
0.0 0.0 0.0
0.0 0.0 0.0
0.0 0.0 0.0
0.0 0.0 0.0
0.0 0.0 0.0
0.0 0.0 0.0
0.0 0.0 0.0
0.0 0.0 0.0
0.0 0.0 0.0
0.0 0.0 0.0
0.0 0.0 0.0
0.0 0.0 0.0
0.0 0.0 0.0
0.0 0.0 0.0
0.0 0.0 0.0
0.0 0.0 0.0
0.0 0.0 0.0
0.0 0.0 0.0
0.0 0.0 0.0
0.0 0.0 0.0

```
0.0 0.0 0.0
0.0 0.0 0.0
0.0 0.0 0.0
0.0 0.0 0.0
0.0 0.0 0.0
0.0 0.0 0.0
0.0 0.0 0.0
0.0 0.0 0.0
0.0 0.0 0.0
0.0 0.0 0.0
0.0 0.0 0.0
0.0 0.0 0.0
0.0 0.0 0.0
0.0 0.0 0.0
0.0 0.0 0.0
0.0 0.0 0.0
0.0 0.0 0.0
0.0 0.0 0.0
0.0 0.0 0.0
remark goes here
MASS
BOND
ANGLE
DIHE
IMPROPER
ca-ca-ca-ha 1.1 180.0 2.0 General improper torsional angle (2
general atom types)
ca-ca-ca-os 1.1 180.0 2.0 Using default value
c2-c3-c2-ha 1.1 180.0 2.0 Using default value
c -ca-ca-ca 1.1 180.0 2.0 Using default value
ca-o -c -o 1.1 180.0 2.0 General improper torsional angle (1
general atom type)
ca-ca-ca-n 1.1 180.0 2.0 Using default value
c -c3-n -ca 1.1 180.0 2.0 Using default value
n -o -c -os 10.5 180.0 2.0 General improper torsional angle (2
general atom types)
NONBOND
```

FORCE FIELD PARAMETER EMPLOYED IN MOLECULAR DOCKING

```
# $Id: AD4.1_bound.dat,v 1.4 2009/04/15 22:38:29 rhuey Exp $
#
# AutoDock
#
# Copyright (C) 1989-2007,  Garrett M. Morris, David S. Goodsell, Ruth Huey, Arthur J. Olson,
# All Rights Reserved.
#
# AutoDock is a Trade Mark of The Scripps Research Institute.
#
# This program is free software; you can redistribute it and/or
# modify it under the terms of the GNU General Public License
# as published by the Free Software Foundation; either version 2
# of the License, or (at your option) any later version.
#
# This program is distributed in the hope that it will be useful,
# but WITHOUT ANY WARRANTY; without even the implied warranty of
# MERCHANTABILITY or FITNESS FOR A PARTICULAR PURPOSE.  See the
# GNU General Public License for more details.
#
# You should have received a copy of the GNU General Public License
# along with this program; if not, write to the Free Software
# Foundation, Inc., 51 Franklin Street, Fifth Floor, Boston, MA  02110-1301, USA.

# AutoDock Linear Free Energy Model Coefficients and Energetic Parameters
#                Version 4.1 Bound
#                 $Revision: 1.4 $

# FE_unbound_model is used to specify how the internal energy of the
```

```
# ligand should be treated when estimating the free energy of binding,
# and can be set to one of the following strings:
#   unbound_same_as_bound, extended, or compact
# unbound_same_as_bound -- this assumes the internal energy of the ligand is the
#                        same before and after binding.
# extended -- this assumes the internal energy of the ligand is that of an
#             extended conformation when unbound.
# compact -- this assumes the internal energy of the ligand is that of a
#            compact conformation when unbound.
#FE_unbound_model unbound_same_as_bound

# AutoDock 4 free energy coefficients with respect to original (AD2) energetic parameters
#  This model assumes that the bound and unbound conformations are the same.
#  See Table 3 in Huey,Morris,Olson&Goodsell (2007) J Comput Chem 28: 1145-1152.
#
#               Free Energy Coefficient
#               ------
FE_coeff_vdW    0.1662
FE_coeff_hbond  0.1209
FE_coeff_estat  0.1406
FE_coeff_desolv 0.1322
FE_coeff_tors   0.2983

# AutoDock 4 Energy Parameters

# - Atomic solvation volumes and parameters
# - Unweighted vdW and Unweighted H-bond Well Depths
#
# - Atom Types
```

```
# - Rii = sum of vdW radii of two like atoms (in Angstrom)
# - epsii = vdW well depth (in Kcal/mol)
# - vol = atomic solvation volume (in Angstrom^3)
# - solpar = atomic solvation parameter
# - Rij_hb = H-bond radius of the heteroatom in contact with a hydrogen (in Angstrom)
# - epsij_hb = well depth of H-bond (in Kcal/mol)
# - hbond = integer indicating type of H-bonding atom (0=no H-bond)
# - rec_index = initialised to -1, but later on holds count of how many of this atom type are in receptor
# - map_index = initialised to -1, but later on holds the index of the AutoGrid map
# - bond_index = used in AutoDock to detect bonds; see "mdist.h", enum {C,N,O,H,XX,P,S}
#
# - To obtain the Rij value for non H-bonding atoms, calculate the
#       arithmetic mean of the Rii values for the two atom types.
#       Rij = (Rii + Rjj) / 2
#
# - To obtain the epsij value for non H-bonding atoms, calculate the
#       geometric mean of the epsii values for the two atom types.
#       epsij = sqrt( epsii * epsjj )
#
# - Note that the Rij_hb value is non-zero for heteroatoms only, and zero for H atoms;
#       to obtain the length of an H-bond, look up Rij_hb for the heteroatom only;
#       this is combined with the Rii value for H in the receptor, in AutoGrid.
#       For example, the Rij_hb for OA-HD H-bonds will be (1.9 + 1.0) Angstrom,
#       and the weighted epsij_hb will be 5.0 kcal/mol * FE_coeff_hbond.
#
#       Atom   Rii                  Rij_hb     rec_index
#       Type       epsii      solpar      epsij_hb   map_index
#                    vol              hbond    bond_index
```

```
#       --     ----  -----  -------  --------  ---  ---  -  --  -- --
atom_par H      2.00  0.020   0.0000   0.00051  0.0  0.0  0  -1  -1  3     # Non H-bonding Hydrogen
atom_par HD     2.00  0.020   0.0000   0.00051  0.0  0.0  2  -1  -1  3     # Donor 1 H-bond Hydrogen
atom_par HS     2.00  0.020   0.0000   0.00051  0.0  0.0  1  -1  -1  3     # Donor S Spherical Hydrogen
atom_par C      4.00  0.150  33.5103  -0.00143  0.0  0.0  0  -1  -1  0     # Non H-bonding Aliphatic Carbon
atom_par A      4.00  0.150  33.5103  -0.00052  0.0  0.0  0  -1  -1  0     # Non H-bonding Aromatic Carbon
atom_par N      3.50  0.160  22.4493  -0.00162  0.0  0.0  0  -1  -1  1     # Non H-bonding Nitrogen
atom_par NA     3.50  0.160  22.4493  -0.00162  1.9  5.0  4  -1  -1  1     # Acceptor 1 H-bond Nitrogen
atom_par NS     3.50  0.160  22.4493  -0.00162  1.9  5.0  3  -1  -1  1     # Acceptor S Spherical Nitrogen
atom_par OA     3.20  0.200  17.1573  -0.00251  1.9  5.0  5  -1  -1  2     # Acceptor 2 H-bonds Oxygen
atom_par OS     3.20  0.200  17.1573  -0.00251  1.9  5.0  3  -1  -1  2     # Acceptor S Spherical Oxygen
atom_par F      3.09  0.080  15.4480  -0.00110  0.0  0.0  0  -1  -1  4     # Non H-bonding Fluorine
atom_par Mg     1.30  0.875   1.5600  -0.00110  0.0  0.0  0  -1  -1  4     # Non H-bonding Magnesium
atom_par MG     1.30  0.875   1.5600  -0.00110  0.0  0.0  0  -1  -1  4     # Non H-bonding Magnesium
atom_par P      4.20  0.200  38.7924  -0.00110  0.0  0.0  0  -1  -1  5     # Non H-bonding Phosphorus
atom_par SA     4.00  0.200  33.5103  -0.00214  2.5  1.0  5  -1  -1  6     # Acceptor 2 H-bonds Sulphur
atom_par S      4.00  0.200  33.5103  -0.00214  0.0  0.0  0  -1  -1  6     # Non H-bonding Sulphur
atom_par Cl     4.09  0.276  35.8235  -0.00110  0.0  0.0  0  -1  -1  4     # Non H-bonding Chlorine
atom_par CL     4.09  0.276  35.8235  -0.00110  0.0  0.0  0  -1  -1  4     # Non H-bonding Chlorine
atom_par Ca     1.98  0.550   2.7700  -0.00110  0.0  0.0  0  -1  -1  4     # Non H-bonding Calcium
atom_par CA     1.98  0.550   2.7700  -0.00110  0.0  0.0  0  -1  -1  4     # Non H-bonding Calcium
atom_par Mn     1.30  0.875   2.1400  -0.00110  0.0  0.0  0  -1  -1  4     # Non H-bonding Manganese
atom_par MN     1.30  0.875   2.1400  -0.00110  0.0  0.0  0  -1  -1  4     # Non H-bonding Manganese
atom_par Fe     1.30  0.010   1.8400  -0.00110  0.0  0.0  0  -1  -1  4     # Non H-bonding Iron
atom_par FE     1.30  0.010   1.8400  -0.00110  0.0  0.0  0  -1  -1  4     # Non H-bonding Iron
atom_par Zn     1.48  0.550   1.7000  -0.00110  0.0  0.0  0  -1  -1  4     # Non H-bonding Zinc
atom_par ZN     1.48  0.550   1.7000  -0.00110  0.0  0.0  0  -1  -1  4     # Non H-bonding Zinc
```

atom_par Br 4.33 0.389 42.5661 -0.00110 0.0 0.0 0 -1 -1 4 # Non H-bonding Bromine

atom_par BR 4.33 0.389 42.5661 -0.00110 0.0 0.0 0 -1 -1 4 # Non H-bonding Bromine

atom_par I 4.72 0.550 55.0585 -0.00110 0.0 0.0 0 -1 -1 4 # Non H-bonding Iodine

atom_par Z 4.00 0.150 33.5103 -0.00143 0.0 0.0 0 -1 -1 0 # Non H-bonding covalent map

atom_par G 4.00 0.150 33.5103 -0.00143 0.0 0.0 0 -1 -1 0 # Ring closure Glue Aliphatic Carbon # SF

atom_par GA 4.00 0.150 33.5103 -0.00052 0.0 0.0 0 -1 -1 0 # Ring closure Glue Aromatic Carbon # SF

atom_par J 4.00 0.150 33.5103 -0.00143 0.0 0.0 0 -1 -1 0 # Ring closure Glue Aliphatic Carbon # SF

atom_par Q 4.00 0.150 33.5103 -0.00143 0.0 0.0 0 -1 -1 0 # Ring closure Glue Aliphatic Carbon # SF
